# Time for Medicine and Public Health to Leave Platform X

**DOI:** 10.2196/53810

**Published:** 2024-05-24

**Authors:** Toomas Timpka

**Affiliations:** 1 Department of Health, Medicine, and Caring Sciences, Linköping University, Linköping, Sweden; 2 Department of Computer and Information Science, Linköping University, Linköping, Sweden; 3 Regional Executive Office, Region Östergötland, Linköping, Sweden

**Keywords:** internet, social media, medical informatics, knowledge translation, digital technology, clinical decision support, health services research, public health, digital health, perspective, medicine

## Abstract

For more than 50 years, digital technologies have been employed for the creation and distribution of knowledge in health services. In the last decade, digital social media have been developed for applications in clinical decision support and population health monitoring. Recently, these technologies have also been used for knowledge translation, such as in the process where research findings created in academic settings are established as evidence and distributed for use in clinical practice, policy making, and health self-management. To date, it has been common for medical and public health institutions to have social media accounts for the dissemination of novel research findings and to facilitate conversations about these findings. However, recent events such as the transformation of the microblog Twitter to platform X have brought to light the need for the social media industry to exploit user data to generate revenue. In this viewpoint, it is argued that a redirection of social media use is required in the translation of knowledge to action in the fields of medicine and public health. A new kind of social internet is currently forming, known as the “fediverse,” which denotes an ensemble of open social media that can communicate with each other while remaining independent platforms. In several countries, government institutions, universities, and newspapers use open social media to distribute information and enable discussions. These organizations control their own channels while being able to communicate with other platforms through open standards. Examples of medical knowledge translation via such open social media platforms, where users are less exposed to disinformation than in general platforms, are also beginning to appear. The current status of the social media industry calls for a broad discussion about the use of social technologies by health institutions involving researchers and health service practitioners, academic leaders, scientific publishers, social technology providers, policy makers, and the public. This debate should not primarily take place on social media platforms but rather at universities, in scientific journals, at public seminars, and other venues, allowing for the transparent and undisturbed communication and formation of opinions.

## Introduction

Digital technologies have been used for the creation and distribution of knowledge in medicine and public health for more than 50 years. In addition to their applications for clinical decision support and population health monitoring [1-5], in the last decade, digital social media have been used for knowledge translation [6] such as in the process of establishing research findings as scientific evidence and distributing these findings for use in clinical practice, policy making, and health self-management. However, in May 2023, an editorial in *Lancet Digital Health*, a leading digital health journal, stated that the social media industry serves as “a commercial determinant of health due to the indirect health consequences of its business practices and actions” [7]. Eight months earlier, the entrepreneur Elon Musk had taken control of the microblog Twitter with a US $44 billion deal. At the time, Twitter was the social media platform most frequently used for knowledge translation and opinion formulation in the fields of medicine and public health. In one of his first actions as Chief Operating Officer, Musk laid off approximately 80% of the company’s employees [8] and phased out most of the content moderation work that countered disinformation and misbehavior [9]. In addition, Musk allowed previously suspended users such as Russian state actors to reactivate their accounts [10]. Changes to the platform also made it essentially impossible for researchers to study the activities occurring on Twitter [11]. Users with verified (paid) accounts were only allowed to read 6000 posts per day, those with unverified accounts could read 600 posts, and new users with unverified accounts could read 300 posts per day. This action exposed that the rationale for the verification and dissemination procedures on the platform is based on financial rather than social capital; consequently, the possibilities for external observers to inspect interactions had been made almost nonexistent. The dismantling of Twitter was manifested by renaming the service X in July 2023, thereby completing the transformation of the platform.

To date, it has been considered standard practice for academic and health service institutions as well as for individual researchers and practitioners to have social media accounts for the dissemination of research findings, access to topical information, and participation in debates about novel discoveries. However, the transformation of Twitter to platform X highlights new threats to the presence of health institutions on social media platforms; in particular, the rational translation of scientific knowledge to health action is currently at stake. In this viewpoint, it is argued that a redirection of social media use is needed with respect to the translation of knowledge to action in medicine and public health.

## Social Media Platforms as Transformative Technologies

More than 60 years ago, mass communication researchers pointed out that the introduction of any novel information technology has the potential to change the shape and character of the affected community [12,13]. Without much forethought or debate, in many countries, social media platforms have become organic components of the information infrastructure. For instance, in Sweden, the proportion of the population regularly following national news broadcasts on the radio and television remained constant between 2009 and 2020, while the proportion of social media users increased from 33% to 78% and the proportion subscribing to printed morning newspaper decreased from 70% to 24% during this same time period [14].

Public institutions active on social media typically choose a platform that they consider generally agrees with the content they want to disseminate; universities and scientific journals share research findings on microblogs such as platform X, whereas cultural authorities tend to use TikTok to share visual content and elementary schools share information in Facebook groups. Even before Twitter was rebranded as platform X, the microblog was associated with several problems and challenges that could have a potential impact on knowledge translation, especially concerning the verification of posted and shared information [15]. The decision made by Elon Musk and his managerial team to reduce the possibility for researchers to analyze the content and interactions occurring on the platform brings to light the fact that a large share of these issues can be traced back to the need for the social media industry to exploit user data to generate revenue.

The logic underpinning such user and customer misuse can be explained by the “life cycle” model of social media platforms [16]. Initially, while financed by seeding financial capital, the platforms produce value for their primary users; the platforms then exploit the users to produce value for business customers and then finally also exploit their business customers to maximize the value for the owners, which eventually leads to death of the platform. The life cycle model thus highlights that small efforts needed to change the operation of social media platforms can serve to rapidly redistribute value between stakeholders in a “two-sided market,” where the platforms sit between users and producers of information. For example, user value is downplayed to produce value for business customers when platform algorithms are tuned to reward conflict-making and fragmentation with the goal of generating more views of posts, which, by extension, increases the display of ads paid for by business customers [17]. A recent study of news dissemination on Facebook [18] reported the greater circulation of conservative than liberal news domains, and indicated that a larger share of the news content was labeled as “false” in the conservative domains than in the liberal domains. The explanation provided by the authors for the faster propagation of conservative content, which has also been observed in other studies [19,20], was that false news stories disseminate faster on social media than true stories because false news items have more “novelty” and tend to arouse more emotions such as fear, disgust, and surprise than true news [21]. The authors argued that the Facebook functions Pages and Groups constitute a news curation and dissemination machine [22], which could then be available to any interest group for manipulation of public opinion [19] or intentionally fracturing an information ecosystem [23].

## The Fediverse and Open Social Media

The problems associated with current social media platforms indicate that if social technologies are to be used for knowledge translation in medicine and public health, this translation should only take place on digital platforms where users are not exploited to create value for the platform’s business partners and investors. The fediverse concept denotes an ensemble of open social media that can communicate with each other while remaining independent platforms [24]. With the emergence of open microblogs such as Mastodon [25] and Bluesky [26], the photo-sharing service Pixelfed [27], and the video service PeerTube [28], users can choose how they want to participate and own their data. Users sign up to specific instances in the fediverse and these instances host their data. The instances are operated by various actors, ranging from the Mastodon project to public institutions, for-profit corporations, nonprofit organizations, and groups of individual users. Technically, the fediverse consists of interconnected network servers running software applications that can read and write the same content.

The open architecture of the fediverse can be compared to that used for email messaging. The email user does not have to compose, organize, and read messages in the same software application, and two users do not have to use the same tools to communicate. The underlying idea is that email simply represents a source of data, and thus many software applications should be able to understand and manipulate these data. In other words, although email applications can have different interfaces, privacy policies, and purposes, every email application can interpret the meaning of an email address and every email address can send messages to every other email address, regardless of the application used. Similarly, if a user posts on Mastodon in the fediverse, another user can see the post in their Pixelfed feed.

The key enabler of the fediverse has been ActivityPub, a communication protocol overseen by the World Wide Web Consortium. More recently, other similar protocols have appeared, including AT managed by Bluesky. In Germany, government institutions, universities, and newspapers have already begun using such forms of open social media [29]. This enables these organizations to control their own channels while still being able to communicate with other platforms through the open standards. In several other countries, many institutions, ranging from public service organizations such as the BBC in England to civil society organizations, have chosen to establish themselves with official accounts at alternative digital platforms. However, reliable data on the magnitude of the migration to alternative digital platforms remain scarce. One early study estimated that approximately 2% of Twitter users deleted their accounts and left the platform for the Mastodon project within the first weeks following the Musk takeover [30]. Approximately 15% of the followers of these users migrated to the exact same Mastodon instance as that of the users they follow. While the larger Mastodon instances attracted more users (the 25% largest instances on Mastodon attract 96% of users), the smaller instances, directed toward specific topics, attracted the more active users.

## Translation of Health Knowledge on Social Media

Based on their responsibility for the peer review, verification, and distribution of research findings, scientific journals play a central role in knowledge translation. Medical and public health journals currently use social media platforms for the promotion and dissemination of content, branding, and facilitating conversation [31,32]. Although the number of social media posts shows a positive correlation with journal Altmetric scores and impact factors [33,34], there is no evidence for causal associations between social media activity and improved knowledge translation. Typically, approximately 40% of all scientific literature is posted on social media [35]; however, half of these posts draw no clicks to the underlying research, whereas an additional 20% of the posts receive only one or two clicks [36]. Moreover, the citation of articles by other researchers will not benefit from social media posting [37]. Instead, the social media presence of scientific journals may indirectly impede rational knowledge translation by luring potential users of new health knowledge to digital environments where research evidence is not necessarily discriminated from unrestrained streams of disinformation. Therefore, medical and public health journals have multiple motivations for reevaluating their social media presence and considering movement to open platforms or even leaving social media altogether. Reconsidering their social media presence is also relevant for the academic and health service institutions that produce and use the knowledge managed by scientific publishers. Establishing accounts and developing the ability to communicate on open microblogs such as Mastodon and Bluesky have become a technically viable alternative along with the use of open standards and protocols such as ActivityPub and AT. By moving to open social media platforms, health institutions can create a digital community that owns and operates their own channels for communication with each other, policy makers, and the public. Open platforms are not susceptible to the capriciousness of private companies and can also provide channels for the dissemination of unaltered health knowledge required during contingencies such as a pandemic. However, moving only one or a few health institutions from platform X to open platforms will not suffice in creating such digital environments.

## Toward a Social Internet for Medicine and Public Health

A new kind of social internet is currently forming. As of February 2024, approximately one-fifth of daily Twitter/X users had left the platform since the Musk takeover in 2022 [38,39]. Considering the status of the social media industry, a short-term goal of medical and public health institutions should begin with contemplating the purpose of their social media presence and explaining how they protect health science beneficiaries from being misled by disinformation (eg, whether and how they promote science literacy [40]) among their followers.

In parallel, a broad discussion is needed about the use of social technologies for knowledge translation in medicine and public health. Examples of translation of medical knowledge in social media platforms where users are less exposed to disinformation are beginning to appear [41]. However, the new social internet also offers possibilities for novel, innovative forms of knowledge translation (eg, in demanding settings such as global health contingencies). For instance, as grounding for a synchronized response to a future pandemic, social media instances with purposive interaction rules [42] can proactively be created in interpandemic periods. Here, instances can be created for separate professional disciplines (from virologists to modelers) and policy-maker categories (from public health officers to politicians) [43]. In parallel, a set of orthogonal instances, organized as multidisciplinary networks, can be created where the professionals and policy makers can collaborate in local and regional response programs [44].

Continued discussion about the use of social media for knowledge translation in medicine and public health should involve researchers and health service practitioners, academic leaders, scientific publishers, social technology providers, policy makers, and the public. This debate should not primarily take place on social media platforms but also at universities, in scientific journals, at public seminars, and other venues, enabling the transparent and undisturbed communication and formation of opinions.
